# Pattern of Prostate Cancer Recurrence Assessed by ^68^Ga-PSMA-11 PET/CT in Men Treated with Primary Local Therapy

**DOI:** 10.3390/jcm10173883

**Published:** 2021-08-29

**Authors:** Ismaheel O. Lawal, Thabo Lengana, Gbenga O. Popoola, Akintunde T. Orunmuyi, Mankgopo M. Kgatle, Kgomotso M. G. Mokoala, Mike M. Sathekge

**Affiliations:** 1 Department of Nuclear Medicine, University of Pretoria, Pretoria 0001, South Africa; ismaheellawal@gmail.com (I.O.L.); tlengana@gmail.com (T.L.); makgopo.kgatle@sanumeri.co.za (M.M.K.); kgomotso.mokoala@up.ac.za (K.M.G.M.); 2 Nuclear Medicine Research Infrastructure (NuMeRI), Steve Biko Academic Hospital, Pretoria 0001, South Africa; 3 Department of Epidemiology and Community Health, University of Ilorin, Ilorin 240102, Nigeria; g.popoola45@gmail.com; 4 Department of Nuclear Medicine, University of Ibadan, Ibadan 200284, Nigeria; akin2nde@gmail.com

**Keywords:** prostate cancer, radical prostatectomy, radiotherapy, biochemical recurrence, PSMA, PET/CT, PSA

## Abstract

Imaging plays a vital role in detecting the recurrence of prostate cancer (PCa) to guide the choice of salvage therapy. Gallium-68 prostate-specific membrane antigen positron-emission tomography/computed tomography (^68^Ga-PSMA-11 PET/CT) is useful for detecting PCa recurrence. We assessed the pattern of PCa recurrence stratified by serum prostate-specific antigen level and type of primary local treatment in men with biochemical recurrence (BCR) after primary local therapy with radical prostatectomy or external beam radiotherapy (EBRT) using ^68^Ga-PSMA-11 PET/CT. We reviewed patients imaged with ^68^Ga-PSMA-11 PET/CT for the localization of the site of PCa recurrence. We determined the site and number of lesions due to PCa recurrence at different PSA levels. A total of 247 men (mean age of 65.72 ± 7.51 years and median PSA of 2.70 ng/mL (IQR = 0.78–5.80)) were included. ^68^Ga-PSMA-11 PET/CT detected the site of recurrence in 81.4% of patients with a median number of lesions per patient of 1 (range = 1–5). ^68^Ga-PSMA-11 PET/CT positivity was 43.6%, 75.7%, 83.3%, 90.0%, and 95.8% at PSA levels of <0.5, 0.5–1.0., 1.1–2.0, 2.1–5.0, and 5.0–10.0, respectively. The most common site of recurrence was in the prostate gland/bed at all PSA levels. Pelvic, extra-pelvic, and combined pelvic and extra-pelvic sites of recurrence were seen in 118, 50, and 33 patients, respectively. The risk of extra-pelvic recurrence increases with rising PSA levels. ^68^Ga-PSMA-11 PET/CT has a high lesion detection rate for biochemical recurrence of PCa in patients previously treated with primary local therapy.

## 1. Introduction

Radical prostatectomy (RP) or external beam radiotherapy (EBRT) is offered to men with organ-confined and locally advanced prostate cancer (PCa) as a definitive primary local treatment [1,2]. Within 10 years of definitive radical treatment, 20–40% of patients treated by RP and 30–50% of patients treated by EBRT will experience disease recurrence [3,4,5]. Disease recurrence, which is heralded by a rise in serum prostate-specific antigen (PSA) levels, is often not accompanied by clinical symptoms. Imaging, therefore, plays a vital role in determining the site of disease recurrence to guide the choice of therapy.

Radiopharmaceuticals targeting cancer cell membrane lipid biosynthesis have been used for positron emission tomography/computed tomography (PET/CT) imaging for the localization of the site of recurrence of PCa. Carbon-11 (^11^C) or Fluorine-18 (^18^F)-labeled acetate or choline are examples of radiopharmaceuticals targeting membrane lipid biosynthesis, which have found widespread application for PET/CT imaging of biochemical recurrence of PCa [6]. These tracers have shown superior sensitivity for the detection of the site of PCa recurrence compared with stand-alone morphologic imaging with magnetic resonance imaging (MRI) or computed tomography (CT) [7]. Despite their excellent performance and widespread application, radiolabeled choline and acetate PET/CT imaging has low sensitivity for disease detection at low PSA levels [8]. Early detection of disease recurrence is crucial to allow for targeted local therapy and defer systemic salvage therapy.

Prostate-specific membrane antigen (PSMA) is overexpressed on the cell membrane of PCa. Small molecule ligands targeting PCa-expressed PSMA have been developed and complex with radionuclides for PET imaging of PCa [9,10]. Many groups have shown the excellent performance of ^68^Ga-PSMA PET/CT imaging for the localization of the site of PCa recurrence [11,12,13,14]. The majority of these studies have focused more on the detection rate of this imaging modality at different serum PSA levels and the factors that influence disease detection rate. Reports documenting the sites of recurrence of PCa in men presenting with biochemical failure are few. Traditionally, salvage radiotherapy to the pelvis is offered to patients with early biochemical recurrence when imaging fails to localize the site of recurrence. This is based on the belief that recurrence of PCa occurs first in the pelvis. It is essential to validate this belief using ^68^Ga-PSMA, a more sensitive imaging modality of PCa recurrence at low serum PSA level. We hypothesize that extra-pelvic recurrence of prostate cancer occurs in patients presenting with early biochemical failure. Therefore, this study aimed to describe the pattern of recurrence of prostate cancer in men presenting with biochemical recurrence (BCR) after primary local therapy with RP or EBRT prior to the institution of salvage therapy. To this end, we stratified the site of recurrence by prior primary local therapy and serum PSA level.

## 2. Materials and Methods

### 2.1. Patients

This is a retrospective study of patients with a prior history of local radical therapy with RP or EBRT for histologically-confirmed PCa referred to the Department of Nuclear Medicine at Steve Biko Academic Hospital, Pretoria, South Africa, between December 2014 and November 2020 for ^68^Ga-PSMA PET/CT imaging to localize the site of recurrence of PCa. Our center introduced ^68^Ga-PSMA PET/CT imaging of PCa in 2014, becoming the first center in Africa to provide this service. Patients were included if they had results of serum PSA obtained within four weeks of imaging and were previously treated with RP or EBRT for histologically confirmed PCa. All the patients included in this study were imaged with ^68^Ga-PSMA-11 PET/CT at the time of confirmation of BCR prior to the institution of salvage therapy. ^68^Ga-PSMA-11 PET/CT was used to select the most suitable salvage therapy in the patients. We excluded patients with prior salvage local or systemic therapy for PCa recurrence, those without recent PSA results (within four weeks before PET/CT imaging), and those treated by other forms of local therapies. We also excluded patients with technically sub-optimal PET/CT images, patients in whom PET/CT was performed with radiopharmaceuticals other than ^68^Ga-PSMA-11, and patients with PSA levels of >10 ng/mL at the time of ^68^Ga-PSMA-11 PET/CT scan. No lower PSA cut-off was applied in patients’ selection as patients were included if serum PSA levels were less than 2 ng/mL plus nadir in patients treated by EBRT or less than 0.2 ng/mL in patients with prior history of RP. All patients provided written consent to undergo ^68^Ga-PSMA PET/CT imaging. The human research ethics committee of the University of Pretoria approved the study and waived the need for patients’ consent due to the retrospective nature of this study.

### 2.2. Synthesis of ^68^Ga-PSMA-11

The synthesis of ^68^Ga-PSMA-11 injected to patients for PET/CT imaging was as we previously reported [15,16]. Briefly, 1 mL of ^68^GaCl_3_ eluted from a ^68^Ge/^68^Ga generator (iThemba LABS, Somerset West, South Africa) was added to a reaction vial containing lyophilized PSMA-11 (ABX advanced biomedical compounds, GmbH, Radeberg, Germany). The solution was incubated at room temperature for 15 min with gentle vortexing. A volume of 1.5 mL of 2.5 M sodium acetate trihydrate and 3 mL of normal saline were added to the solution to obtain a physiologic pH. Radiochemical purity was above 95% in all synthesized radiopharmaceutical injected into the patients included in this study.

### 2.3. ^68^Ga-PSMA PET/CT Imaging

We performed no special patient preparation prior to ^68^Ga-PSMA-11 PET/CT imaging. The activity of ^68^Ga-PSMA-11 administered was corrected for weight. An amount of 2 MBq/Kg body weight was injected. Imaging commenced after a 60 min uptake time. Whole-body imaging (vertex to mid-thigh) was performed on a Biograph 40 TruePoint hybrid PET/CT scanner (Siemens Medical Solution, Chicago, IL, USA). Frusemide or intravenous contrast was not routinely used for imaging. The details of the imaging parameters used are as previously published [17,18].

### 2.4. Image Analysis and Data Collection

Two board-certified nuclear medicine physicians, each with 7 years of experience reading ^68^Ga-PSMA-11 PET/CT scans, performed image interpretation independently. A third board-certified nuclear medicine physician resolved differences in interpretation between the two expert readers with similar years of experience. Image analysis was performed on a dedicated workstation equipped with a Syngo.via software (Siemens Medical Solution, Chicago, IL, USA). CT-identified lesions with increased ^68^Ga-PSMA-11 uptake above background activity level that do not conform to physiologic uptake or known pitfalls of ^68^Ga-PSMA-11 PET/CT imaging were interpreted as positive for the site of PCa recurrence [19]. For each patient, the sites of PCa recurrence and the number of lesions were documented. We stratified the site of recurrence as pelvic if recurrence of PCa was localized to the prostate gland/prostate bed, pelvic lymph nodes, or any other pelvic soft tissue. Recurrence at other sites was stratified as extra-pelvic sites of recurrence.

We reviewed the medical records of all included patients to extract relevant disease-related information, including demographic data, serum PSA level within four weeks before ^68^Ga-PSMA-11 PET/CT scan, Gleason score, ISUP (International Society of Urological Pathology) grade group, and the type of local radical therapy received.

### 2.5. Statistical Analysis

We performed descriptive statistics of the demographic and baseline disease-related characteristics. We used the Chi-square test to compare the disease-related characteristics in patients with positive versus those with negative ^68^Ga-PSMA-11 PET/CT scans. We also compared baseline disease-related characteristics between patients treated by RP versus EBRT using the Chi-square test, Fisher’s Exact test, Independent Samples T-test, and Mann–Whitney U test as appropriate. We stratified patients in whom ^68^Ga-PSMA-11 PET/CT was positive for the site of prostate cancer recurrence according to their serum PSA levels and assessed for any significant difference in the number lesions detected using Kruskal–Wallis test. We compared patients with pelvic versus extra-pelvic site of PCa recurrence according to their baseline disease-related characteristics using the Chi-square test. We set statistical significance at a *p* value of <0.05. We used IBM SPSS Statistics (IBM Corp., Armonk, NY, USA) for statistical analysis.

## 3. Results

A total of 247 men with biochemical recurrence of PCa following radical local therapy with either RP or EBRT without prior salvage therapy were included in this study (Supplementary Figure). The mean age of the study population was 65.72 ± 7.51 years (range = 47–88 years). At the time of ^68^Ga-PSMA-11 PET/CT imaging, the median PSA level was 2.70 ng/mL (interquartile range, IQR = 0.78–5.80) with a modal ISUP grade group of 1 (range = 1–5). Among the study population, 157 patients (63.60%) had primary local treatment with RP, while 90 patients (36.40%) were previously treated with EBRT. ^68^Ga-PSMA-11 PET/CT identified the site of recurrence in 201 patients with an imaging positivity rate of 81.4% and a median number of sites of recurrence of 1 (range = 1–5). Table 1 shows the details regarding demography, baseline disease-related characteristics, and the ^68^Ga-PSMA PET/CT positivity rate of the study population.

### 3.1. Pattern of Prostate Cancer Recurrence

**Figure 1:**
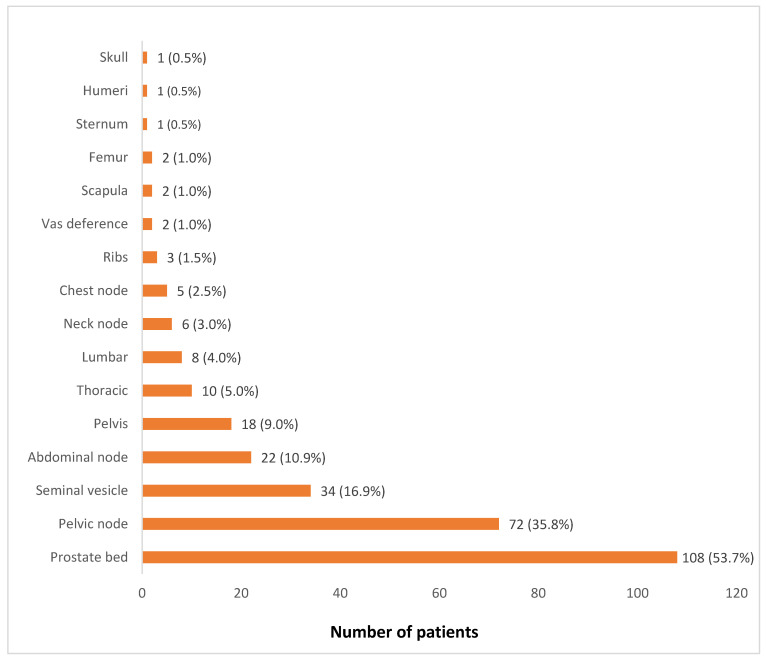
Distribution of the sites of recurrence of prostate cancer.

Figure 1 shows the distribution of sites of recurrence of PCa. The prostate gland/prostate bed was the commonest site of PCa recurrence seen in more than half of the patients with a positive ^68^Ga-PSMA-11 PET/CT scan (53.7%). Loco-regional recurrence in pelvic lymph nodes and seminal vesicles were seen in 35.5% and 16.9% of patients, respectively. No patient had a visceral site of PCa recurrence. Among the patients that showed skeletal sites of recurrence, the pelvic bones (9.0%), thoracic spine (5.0%), and lumbar spine (4.0%) were the most common sites of skeletal metastatic recurrence. Table 2 shows the number of lesions among patients with a positive ^68^Ga-PSMA PET/CT finding for PCa recurrence stratified according to serum PSA level. Higher serum PSA level was significantly associated with a higher number of PCa recurrent lesions (*p* = 0.003).

**Figure 2:**
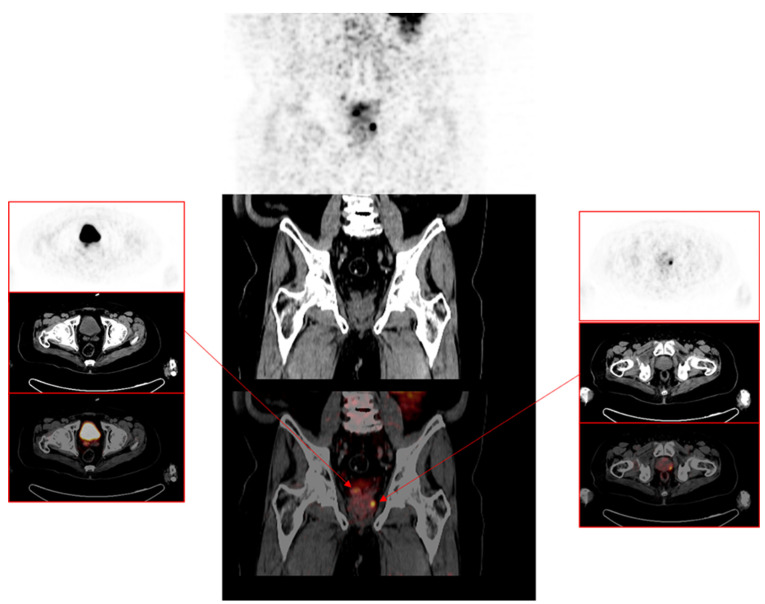
A 77-year-old male previously treated with external beam radiotherapy for adenocarcinoma of the prostate, Gleason 4+4. ^68^Ga-PSMA-11 PET/CT was requested for the localization of disease recurrence at serum PSA of 0.61 ng/mL. Central images are the coronal PET-only, CT-only, and fused PET/CT images of the pelvis, localizing disease recurrence to the right seminal vesicle (right insert) and a focal lesion in the left lobe of the prostate gland (left insert).

**Figure 3:**
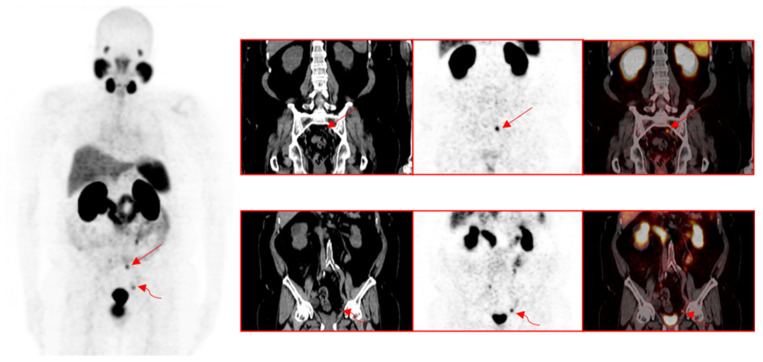
A 63-year-old male with a history of radical prostatectomy for adenocarcinoma of the prostate, Gleason 4+4. Ga-PSMA-11 PET/CT was requested for localization of disease recurrence at a serum PSA of 0.54 ng/mL. Images show intense tracer uptake in a subcentimeter left presacral node (straight arrows) and a subcentimeter left external iliac node consistent with the sites of prostate cancer recurrence.

**Figure 4:**
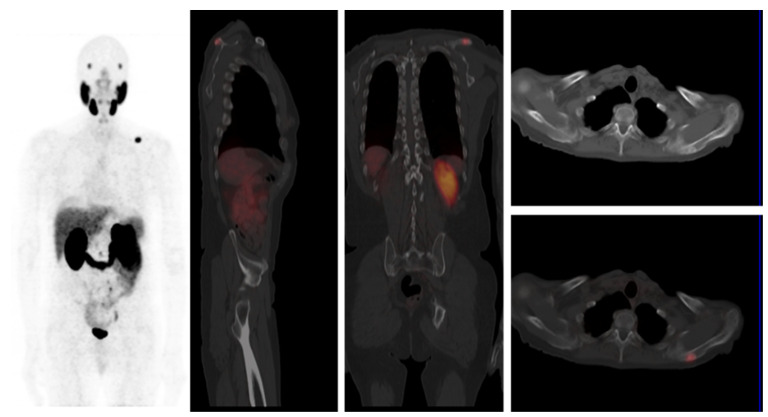
A 68-year-old male known with adenocarcinoma of the prostate, Gleason 4+3. He had radical prostatectomy for local radical therapy. Ga-PSMA-11 PET/CT was obtained at serum PSA level of 3.46 ng/mL to localize the site of disease recurrence. Images obtained from his scan show intense tracer uptake in the left scapula consistent with a solitary skeletal site of extra-pelvic recurrence of prostate cancer. Multiple focal areas of increased tracer accumulation seen in the maximum intensity image (right image) on the left side of the midline in the abdominopelvic area are consistent with urine within the left ureter.

Out of 201 patients with a positive ^68^Ga-PSMA-11 PET/CT finding for recurrence of PCa, 118 patients (58.7%) had a pelvic site of recurrence, 50 patients (24.9%) had an extra-pelvic site of recurrence, while 33 patients (16.4%) had PCa recurrence localized to pelvic and extra-pelvic sites. Pelvic recurrence predominated at all serum PSA levels. The highest risk of combined pelvic and extra-pelvic PCa recurrence occurred at the highest serum PSA group (5.1–10.0 ng/mL). While patients with prior treatment with EBRT were more likely to have a pelvic recurrence, they were also more likely than patients previously treated with RP to have combined pelvic and extra-pelvic sites of disease recurrence (Table 3). The ISUP grade group was not significantly different between patients stratified according to the type of primary radical therapy. Figure 2, Figure 3 and Figure 4 are representative images from our study cohort.

We found significant differences in the group of patients treated with RP versus patients treated with EBRT regarding disease-related characteristics and the site of PCa recurrence. For example, patients previously treated with RP had a significantly lower serum PSA at the time of assessment compared with patients with a prior history of EBRT (1.77 vs. 4.35 ng/mL, *p* < 0.001). Consequently, patients previously treated with EBRT were more likely to have positive findings for recurrence (95.6%) compared with patients previously treated with RP (73.2%), *p* < 0.001. Patients previously treated with EBRT were more likely to have a recurrence in the prostate gland compared with patients previously treated with RP, *p* < 0.001. Table 4 shows a detailed comparison between patients stratified by the type of radical primary therapy regarding their baseline disease-related characteristics and sites of PCa recurrence.

### 3.2. Comparison of Patients with Positive versus Negative Findings for Recurrence on ^68^Ga-PSMA-11 PET/CT

No significant difference in age of patients with positive ^68^Ga-PSMA-11 PET/CT findings versus patients in whom imaging failed to localize the site of PCa recurrence (*p* = 0.078) was found. ISUP grade grouping was also similar between the two groups of patients. On the contrary, patients with a positive ^68^Ga-PSMA-11 PET/CT finding for recurrence had higher serum PSA (3.22 ng/mL) versus patients with a negative finding (0.51 ng/mL). Similarly, there was a significantly higher proportion of patients previously treated with EBRT who had positive ^68^Ga-PSMA-11 PET/CT finding (95.6%) compared with patients previously treated by RP (73.2%), *p* < 0.001. Lesion detection rate by ^68^Ga-PSMA-11 PET/CT increased with increasing serum PSA level (Table 5). Ten patients who were previously treated with RP had ^68^Ga-PSMA-11 PET/CT performed at serum PSA level below 0.2 ng/mL. Of these 10 patients, at least one site of PCa recurrence was seen in three patients (30% positivity rate). Table 5 shows the detailed comparison between patients with and without positive finding suggestive of PCa recurrence on ^68^Ga-PSMA-11 PET/CT imaging.

## 4. Discussion

Several studies have reported excellent detection rates for ^68^Ga-PSMA-11 PET/CT in assessing BCR of PCa [8,11,12,14,20]. Most of these studies have focused on the disease detection rate of ^68^Ga-PSMA-11 PET/CT in patients with BCR of PCa. Several studies have evaluated the utility of ^68^Ga-PSMA-11 PET/CT to assess the pattern of recurrence of PCa [21,22,23,24,25,26]. These studies were mostly focused on patients with early BCR at PSA of <2.0 ng/mL. With metastasectomy being increasingly offered to patients with oligometastatic recurrence of PCa, a need exists to assess the diagnostic performance of ^68^Ga-PSMA-11 PET/CT in patients with higher PSA levels (up to 10 ng/mL) who may have a more widespread pattern of recurrence. In this study, we comprehensively assessed the recurrence pattern of PCa on ^68^Ga-PSMA-11 PET/CT in 247 men presenting with BCR at a PSA level of <10.0 ng/mL before the institution of salvage therapy. We stratified the site of recurrence by prior primary local therapy and serum PSA level to determine the impact of these variables on PCa recurrence. We found a ^68^Ga-PSMA-11 PET/CT positivity rate of 81.4%. BCR lesion detected per patient ranged from 1 to 5 lesions (median = 1). As with previous studies, ^68^Ga-PSMA-11 PET/CT positivity rate demonstrated a directly proportional relationship with serum PSA. Detection rates were 43.6%, 75.7%, 83.8%, 90.0%, and 95.8% at PSA levels of <0.5, 0.5–1.0, 1.1–2.0, 2.1–5.0, and 5.1–10.0 ng/mL, respectively. These detection rates are within the published ranges of the diagnostic sensitivity ^68^Ga-PSMA PET/CT for BCR of PCa [20].

Regardless of the type of primary radical therapy, ISUP grade group, or serum PSA level, the pelvic soft tissues (prostate gland/prostate bed and pelvic lymph nodes) were the most common sites of disease recurrence. A total of 118 patients (47.8% of the study population) had recurrence localized to the pelvic region, while 33 patients (13.4%) had combined pelvic and extra-pelvic sites of PCa recurrence. Recurrence within the prostate gland/bed seen in 108 patients was significantly more prevalent among post-EBRT patients compared with post-RP patients. This higher prostate gland recurrence rate in the post-EBRT patient is congruent with the differential pooled recurrence rate between patients previously treated with EBRY versus RT, reported by Perera and colleagues in their recent meta-analysis [20]. Similar to the pooled incidence of recurrence in the different extra-prostate/prostate bed sites in the meta-analysis by Perera et al., we did not find any significant difference in the pattern of extra-prostatic sites of recurrence between patients previously treated with EBRT versus RP [20]. In the absence of histological confirmation of recurrence, an assessment of prostate gland recurrence based on ^68^Ga-PSMA uptake on PET/CT imaging must be approached with caution. In the study by Fendler et al., a common cause of false-positive ^68^Ga-PSMA PET/CT findings in patients imaged for BCR of PCa was due to focal intense tracer uptake in the prostate gland, which was confirmed on histological evaluation to be benign prostate tissue with strong expression of PSMA [19].

Salvage radiotherapy is most successful for biochemical response when offered at a low serum PSA level. There is a 2.6% loss of biochemical control for every 0.1 ng/mL rise in PSA level [27]. Several patients offered salvage radiotherapy at low serum PSA may fail to achieve complete biochemical control, most likely due to the coexistence of extra-pelvic metastatic recurrence. In our cohort, we found an incremental prevalence of extra-pelvic sites of recurrence with a rising serum PSA level. There were 7 (2.4%), 8 (3.2%), 7 (2.8%), 22 (8.9%), and 39 (15.8%) patients with extra-pelvic only or combined pelvic and extra-pelvic sites of PCa recurrence at serum PSA levels of <0.5, 0.5–1.0, 1.1–2.0, 2.1–5.0, and 5.1–10.0 ng/mL, respectively. This finding supports applying whole-body imaging with a sensitive modality like ^68^Ga-PSMA-11 PET/CT rather than limited pelvic imaging such as pelvic MRI in the work-up of patients with BCR even at low PSA level. Similar to the site of PCa recurrence, we also found an increasing number of lesions due to PCa recurrence with a rising serum PSA level. At low PSA levels below 0.5 ng/mL, lesions due to PCa recurrence ranged from 1–2, with a median of 1 lesion. At PSA levels above 2 ng/mL, lesions due to PCa recurrence detected on ^68^Ga-PSMA PET/CT ranged from 1 to 5.

In comparing the 201 patients with ^68^Ga-PSMA-11 PET/CT positivity for PCa recurrence versus the 46 patients with a negative imaging finding, we found no significant difference regarding the age and ISUP grade grouping between the two groups. The median serum PSA level was significantly higher for the ^68^Ga-PSMA PET/CT positivity group compared with the negative group. Patients who had EBRT as their primary radical mode of therapy were more likely to have positive findings for BCR on ^68^Ga-PSMA PET/CT than patients previously treated with RP. This significant difference in ^68^Ga-PSMA PET/CT positivity according to the type of primary radical therapy is most likely related to the differences in serum PSA between the two groups. The median PSA at the time of ^68^Ga-PSMA PET/CT was 4.35 ng/mL (IQR = 1.92–7.60) in the EBRT compared with 1.77 ng/mL (IQR = 0.53–4.26) in the RP group, *p* < 0.001.

Our study includes many strengths, including the large study population. Our patient population is representative of the patients presenting with BCR in Sub-Saharan Africa, as our center was the first to introduce ^68^Ga-PSMA PET/CT service on the continent. When we were the only center providing this service during the early years, we received referrals from across and beyond South Africa, including the West African, Central African, East African, and the Southern African subregions. We included a broader range of patients in terms of serum PSA levels and the mode of primary radical therapy. Our results, therefore, complement the results of studies that have reported a narrower range of patients. For example, our results show the performance of ^68^Ga-PSMA PET/CT in evaluating the pattern of recurrence at higher PSA levels and in patients previously treated with EBRT. In this study, we included only patients who have not had salvage therapy of any form at the time of imaging. This is a significant departure from previous studies that included mixed populations of treatment-naive patients and patients with prior history of salvage therapy (radiotherapy or androgen-deprivation therapy) for BCR of PCa in their study cohorts. Our study also includes important limitations, one of which is its retrospective design. Similar to many studies on this subject, we did not have histological confirmation of all lesions assessed as PCa recurrence on ^68^Ga-PSMA-11 PET/CT. Despite its lack of specificity, ^68^Ga-PSMA PET/CT still has a high positive predictive value when applied in the right setting [19]. Highly experienced physicians performed image interpretation. This would have contributed to avoiding known pitfalls that have been identified to compromise the accurate interpretation of ^68^Ga-PSMA-11 PET/CT for BCR assessment. We did not include data on the impact of ^68^Ga-PSMA-11 PET/CT imaging on treatment management decisions or survival. These data are not routinely collected in our practice. In selecting patients for this study, we did not apply the strict criteria for the definition of biochemical recurrence according to the Phoenix criteria [28]. This implied that we included patients with low PSA who might not have qualified to be investigated for BCR. This is generally in line with the contemporary practice in which patients are now investigated for BCR before reaching the threshold recommended in guidelines that predate the era of ^68^Ga-PSMA-11 PET/CT for BCR recurrence [29].

## 5. Conclusions

^68^Ga-PSMA-11 PET/CT has a high lesion detection rate for biochemical recurrence of prostate cancer at a serum PSA level of <10.0 ng/mL in patients previously treated with radical prostatectomy or external beam radiotherapy. The number of lesions due to recurrence and extra-pelvic sites of recurrence increase with an increase in serum PSA. ^68^Ga-PSMA-11 PET/CT will be useful to guide the choice of salvage therapy in patients with PCa recurrence.

## Figures and Tables

**Table 1:** Demography, baseline disease-related characteristics, and ^68^Ga-PSMA-11 PET/CT positivity rate.

Variable	Frequency	Percent
PSA (ng/mL)		
<0.5	39	15.8
0.5–1.0	37	15.0
1.1–2.0	30	12.1
2.1–5.0	70	28.3
5.1–10.0	71	28.7
Median (IQR)	2.70 (0.78–5.80)	
Range	0.05–9.91	
ISUP grade group		
1	68	27.5
2	55	22.3
3	46	18.6
4	30	12.1
5	21	8.5
NA	27	10.9
Median (IQR)	2 (1–5)	
Range	1–5	
Primary therapy		
Radical prostatectomy	157	63.6
EBRT	90	36.4
^68^Ga-PSMA PET-positive for recurrence		
Yes	201	81.4
No	46	18.6
Number of lesions		
Median (IQR)	1 (1–2)	
Range	1–5	

PSA: prostate-specific antigen; ISUP: International Society of Urological Pathology; NA: patients with missing Gleason score; EBRT: external beam radiotherapy.

**Table 2:** Relationship between PSA level and number of lesions among patients with positive ^68^Ga-PSMA-11 PET/CT finding for recurrence.

PSA (ng/mL)	*n*	Median (IQR)	Range	K	*p* Value
<0.5	17	1.0 (1.0)	1.0–2.0	16.234	0.003
0.5–1.0	28	1.0 (1.0)	1.0–3.0		
1.1–2.0	25	1.0 (1.0–1.5)	1.0–4.0		
2.1–5.0	63	1.0 (1.0–2.0)	1.0–5.0		
5.1–10.0	68	1.0 (1.0–2.0)	1.0–5.0		

K: Kruskal–Wallis test; PSA: prostate-specific antigen.

**Table 3:** Site of recurrence stratified according to ISUP grade group, PSA level, and prior primary therapy.

	Site of recurrence on ^68^Ga-PSMA-11 PET/CT				
	Pelvic	Extra-Pelvic	Both	ꭓ^2^	*p* Value
Variable	*n* (%)	*n* (%)			
ISUP grade group					
1	32 (57.1)	16 (28.6)	8 (14.3)	15.015	0.059
2	27 (67.5)	11 (27.5)	2 (5.0)		
3	18 (47.4)	13 (34.2)	7 (18.4)		
4	17 (65.4)	1 (3.8)	8 (30.8)		
5	11 (57.9)	4 (21.1)	4 (21.4)		
PSA (ng/mL)					
<0.5	10 (58.8)	6 (35.3)	1 (5.9)	19.602	0.012
0.5–1.0	20 (71.4)	6 (21.4)	2 (7.1)		
1.1–2.0	18 (72.0)	5 (20.0)	2 (8.0)		
2.1–5.0	41 (65.1)	15 (23.8)	7 (11.1)		
5.1–10.0	29 (42.6)	18 (26.5)	21 (30.9)		
Primary therapy					
Radical prostatectomy	64 (55.7)	37 (32.2)	14 (12.2)	9.131	0.010
EBRT	54 (62.8)	13 (15.1)	19 (22.1)		

ꭓ^2^: Chi-square test; PSA: prostate-specific antigen; ISUP: International Society of Urological Pathology; EBRT: external beam radiotherapy.

**Table 4:** Comparison of patients stratified according to the type of primary radical therapy.

	Primary Therapy			
	Radical Prostatectomy	Radiotherapy	ꭓ^2^	*p* Value
Variable	*n* (%)	*n* (%)		
Age (years)				
Mean ± SD	65.39 ± 7.52	66.28 ± 7.49	−0.889 ^t^	0.375
Range	47–88	51–87		
PSA (ng/mL)				
<0.5	33 (21.0)	6 (6.7)	25.770	<0.001
0.5–1.0	26 (16.6)	11 (12.2)		
1.1–2.0	25 (15.9)	5 (5.6)		
2.1–5.0	42 (26.8)	28 (31.1)		
5.1–10.0	31 (19.7)	40 (44.4)		
Median (IQR)	1.77 (0.53–4.26)	4.35 (1.92–7.60)	4447.500 ^U^	<0.001
Range	0.05–9.90	0.05–9.91		
ISUP grade group				
1	31 (45.6)	37 (54.4)	13.816	0.008
2	41 (74.5)	14 (25.5)		
3	33 (71.7)	13 (28.3)		
4	20 (66.7)	10 (33.3)		
5	14 (66.7)	7 (33.3)		
Median (IQR)	2 (2–3)	2 (1–3)	4519.000 ^U^	0.012
Range	1–5	1–5		
Recurrence				
Yes	115 (73.2)	86 (95.6)	18.783	<0.001
No	42 (26.8)	4 (4.4)		
Site of recurrence **				
Prostate bed/gland	50 (31.8)	58 (64.4)	24.704	<0.001
Seminal vesicle	19 (12.1)	15 (16.7)	1.004	0.316
Vas deference	1 (0.6)	1 (1.1)	0.160 ^F^	1.000
Pelvic node	40 (25.5)	32 (35.6)	2.813	0.093
Abdominal node	14 (8.9)	8 (8.9)	<0.001	0.994
Chest node	3 (1.9)	2 (2.2)	0.028 ^F^	1.000
Neck node	5 (3.2)	1 (1.1)	1.038 ^F^	0.421
Pelvic bone	10 (6.4)	8 (8.9)	0.537	0.463
Lumbar spine	4 (2.5)	4 (4.4)	0.657 ^F^	0.467
Thoracic spine	8 (5.1)	2 (2.2)	1.216 ^F^	0.335
Rib	2 (1.3)	1 (1.1)	0.013 ^F^	1.000
Scapular	2 (1.3)	0 (0.0)	1.156 ^F^	0.535
Clavicle	1 (0.6)	0 (0.0)	0.576 ^F^	0.448
Sternum	1 (0.6)	0 (0.0)	0.576 ^F^	1.000
Humerus	1 (0.6)	0 (0.0)	0.576 ^F^	1.000
Femur	2 (1.3)	0 (0.0)	1.156 ^F^	0.535
Skull	0 (0.0)	1 (1.1)	1.752 ^F^	0.186

**: Multiple sites of recurrence present in certain patients; ꭓ^2^: Chi-square test; F: Fisher’s exact test; t: independent samples T-test; U: Mann–Whitney U test; PSA: prostate-specific antigen; ISUP: International Society of Urological Pathology; EBRT: external beam radiotherapy.

**Table 5:** Comparison of patients with positive ^68^Ga-PSMA-11 PET/CT finding versus patients with negative finding for prostate cancer recurrence.

	^68^Ga-PSMA PET Positivity for Recurrence			
	Yes	No	ꭓ^2^	*p* Value
Variable	*n* (%)	*n* (%)		
Age (years)				
Mean ± SD	66.12 ± 7.38	63.96 ± 7.89	1.770 ^t^	0.078
Range	48–88	47–85		
PSA (ng/mL)				
<0.5	17 (43.6)	22 (56.4)	50.760	<0.001
0.5–1.0	28 (75.7)	9 (24.3)		
1.1–2.0	25 (83.3)	5 (16.7)		
2.1–5.0	63 (90.0)	7 (10.0)		
5.1–10.0	68 (95.8)	3 (4.2)		
Median (IQR)	3.22 (1.21–6.58)	0.51 (0.32–1.79)	2066.000 ^U^	<0.001
Range	0.05–9.91	0.05–7.64		
ISUP grade group				
1	56 (82.4)	12 (17.6)	4.503	0.342
2	40 (72.7)	15 (27.3)		
3	38 (82.6)	8 (17.4)		
4	26 (86.7)	4 (13.3)		
5	19 (90.5)	2 (9.5)		
Median (IQR)	2 (1–4)	2 (1–3)	3323.000 ^U^	0.332
Range	1–5	1–5		
Primary therapy				
Radical prostatectomy	115 (73.2)	42 (26.8)	18.783	<0.001
EBRT	86 (95.6)	4 (4.4)		

ꭓ^2^: Chi-square test; t: independent samples T-test; U: Mann–Whitney U test; PSA: prostate-specific antigen; ISUP: International Society of Urological Pathology; EBRT: external beam radiotherapy.

## Data Availability

Data upon which the results presented in this study are available upon reasonable request from the corresponding author.

## Supplementary Materials

The following are available online at https://www.mdpi.com/article/10.3390/jcm10173883/s1, Figure S1: A flowchart showing the selection of patients for the study.
