# Supplementary figures and images for: Pattern of Prostate Cancer Recurrence Assessed by 68Ga-PSMA-11 PET/CT in Men Treated with Primary Local Therapy
Source: J Clin Med. 2021 Aug 29;10(17):3883. doi: 10.3390/jcm10173883 (PMC8432125; doi:10.3390/jcm10173883)

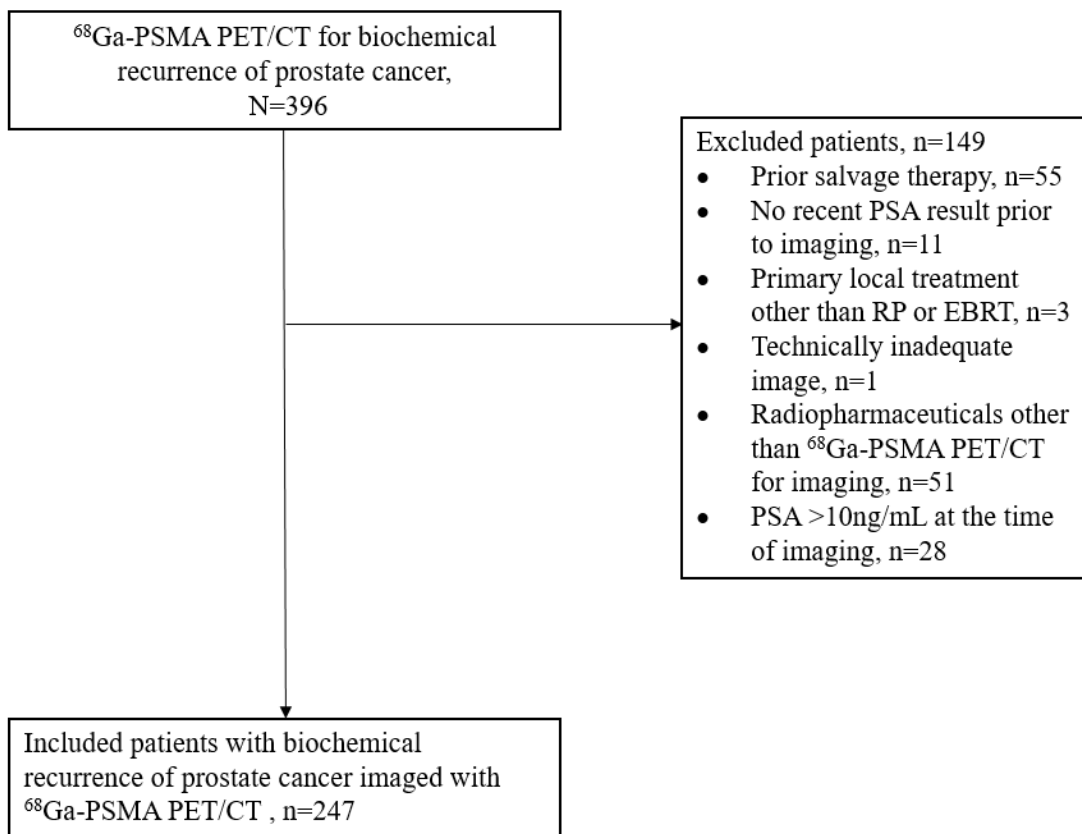

Figure S1: A flowchart showing the selection of patients for the study.

Supplement: Supplementary file 1 [file jcm-10-03883-s001.zip › jcm-1297351-supplementary.pdf]
